# Effect of infrared lamps to ameliorate cold stress in Vrindavani calves

**DOI:** 10.14202/vetworld.2015.777-782

**Published:** 2015-06-24

**Authors:** Showkat A. Bhat, Bharat Bhushan, Sajad A. Sheikh, T. Chandrasekar, Asu Singh Godara, Pranay Bharti, K. Puhle Japheth

**Affiliations:** 1 Livestock Production and Management Section, National Dairy Research Institute, Karnal, Haryana, India; 2 Division of Animal Genetics, Indian Veterinary Research Institute, Izatnagar, Uttar Pradesh, India; 3 Division of Poultry Science, Central Avian Research Institute, Izatnagar, Uttar Pradesh, India; 4 Livestock Production and Management Section, Indian Veterinary Research Institute, Izatnagar, Uttar Pradesh, India

**Keywords:** body weight, cold stress, infrared lamps, Vrindavani calves

## Abstract

**Aim:**

This study was conducted to determine the effect of infrared lamps to ameliorate cold stress in Vrindavani (Holstein Friesian × Brown Swiss × Jersey × Hariana) calves.

**Materials and Methods:**

For the present investigation, ten newborn Vrindavani calves were randomly divided into two groups (G_1_ and G_2_) of five each. The experiment was conducted from 2^nd^ November to 8^th^ February when the environmental temperature was at the lowest. The calves of G_1_ were provided with no additional protection while the calves of G_2_ were protected against the cold weather by providing heat using the infrared lamps. The body weight (kg) of the calves was recorded at weekly interval. The blood samples collected within 6 h of birth and then at fortnightly interval were analyzed for packed cell volume (PCV, %), hemoglobin (Hb, g/dl). Besides, the serum biochemical parameters, *viz*., Total serum protein (TSP, g/l), albumin (g/l), globulin (g/l), albumin globulin ratio (A:G) and important stress parameters, *viz*., triiodothyronine (T_3_, ng/ml), thyroxine (T_4_, ng/ml) and cortisol (ng/ml) were also estimated.

**Results:**

The calves of G_2_ showed higher body weight gain as compared to G_1_. The differences were found to be highly significant (p<0.01). The calves in G_1_ showed comparatively higher values of PCV and Hb and the differences were found to be significant (p<0.05) on 45^th^ day for PCV and highly significant (p<0.01) on 60^th^ day for PCV and on 45^th^ day for Hb. The values of TSP and albumin were comparatively higher in calves of G_1_ as compared to G_2_ and the differences were highly significant (p<0.01) on 45^th^ day for both TSP and albumin and significant (p<0.05) on 60^th^ day for albumin. Significantly (p<0.01) higher values of cortisol and T_4_ were observed on 15 and 45^th^ day in calves of G_1_ as compared to G_2_. The T_3_ levels were also found higher in calves of G_1_ than G_2_ and the differences were significant (p<0.05) on 15 and 30^th^ day and highly significant (p<0.01) on 45^th^ day of the study.

**Conclusion:**

Based on the results, it could be concluded that the infrared lamps are efficient in providing favorable microclimate and hence can be effectively used in calf shed to protect newborn calves from adverse conditions of winter and to improve their body growth performance.

## Introduction

The success of any dairy enterprise depends on the successful raising of the young calves. Increasing the proportion of calves that survive to weaning is of utmost economic importance. Calf mortality is a major concern at both farmers’ level as well as at organized farm. Pre-weaning mortality is an important economic component of farming system since it reduces the number of animals available for sale [1], compromises animal well-being [2] and reduces the number of animals available for selection as well as genetic progress [3,4]. Extreme variations in the ambient temperature can influence the growth of calves to a greater extent and prolonged exposure may lead to stunting of the calves along with a compromised immune status. Calves and lambs are relatively cold sensitive at birth due to their relatively larger surface area than adults, lack of heat production from rumen fermentation and being wet from fetal fluid [5]. The relative constant conditions that exist within the body of a healthy animal are achieved by operation of innumerable homeostatic control measures [6]. During early post-natal life, homeotherms undergo marked developmental changes to control their body temperature [7]. Apart from fluctuations in the physiological and biochemical profiles, T_3_ and T_4_ also play a major role in thermogenesis [8], especially when animals are exposed to cold stressful situations [9]. During winter, as energy intake is used mainly for thermoregulation therefore, it is possible to observe a depression in body weight gain and an increase in mortality [10].

The survival of a calf and its optimum growth rate can be achieved not only by good feeding, but also with efficient management compatible with specific environmental conditions. This basically becomes more important when seasonal variations are quite wide and extreme. The thermoneutral zone for calves lies in a range of 15-25°C [11] and the lower critical temperature ranges from 9°C to 15°C at birth and during the first 2 weeks of life [12]. It appears reasonable for the livestock sector to come up with new strategies in order to maintain and increase the production potential under altered climatic conditions. Several effective measures, including the use of calf jackets, hot box or warm water bath are used to prevent cold stress [13]. Reduction in the overall heating requirement of calf shed can be accomplished by taking advantage of the thermal and optical properties of infrared radiations. Due to the directional property of thermal radiations, the radiant energy can be transmitted directly from the source of radiation to calves.

The present study was, therefore, undertaken with the aim to observe the effect of infrared lamps to ameliorate cold stress in crossbred Vrindavani calves.

## Materials and Methods

### Ethical approval

This study was conducted after approval by the Research Committee and Institutional Animal Ethics Committee of Indian Veterinary Research Institute.

### Climatic conditions and experimental animals

The experiment was carried out at the calf unit of Cattle and Buffalo Farm, Livestock Production Management Section, Indian Veterinary Research Institute, Izatnagar, Bareilly, Uttar Pradesh (India) which is located at an altitude of 169 meters above mean sea level and at the latitude of 28.22 °N and longitude of 79.22 °E. The climate of the place touches both the extremes of hot (approximately 45°C) and cold (approximately 5°C) and Relative Humidity ranges between 15% and 99%. The average maximum and minimum values of air temperature during the last 3 years (2011, 2012 and 2013) were 38.07°C and 7.43°C, respectively. During study period mean environmental temperature and relative humidity ranged between 9.5°C and 21.5°C and 72.1% and 91.7%, respectively inside the calf shed. The external temperature and relative humidity varied between 7°C to 18°C and 58% to 95%, respectively.

The experiment was conducted from 2^nd^ November, 2013 to 8^th^ February, 2014 when the environmental temperature was at the lowest. Ten newborn Vrindavani (Friesian × Brown Swiss × Jersey × Hariana) calves were randomly divided into two Groups (G_1_) and (G_2_) of five each. Less number of calves was available for the study due to low calving rate at the farm during winter. All the calves were kept in the individual calf pens. Calves of G_1_ were provided with no heat source while the calves of G_2_ were provided protection against the cold weather through the use of 250 Watt infrared lamps. Infrared lamps were used at the rate of one per two calves placed at a height of 30 inches from the body of the calf. The infrared lamps were used from 500 p.m. to 900 a.m. in order to protect the calves from adverse effects of cold weather. Both the groups were provided paddy straw bedding in their respective pens. The bedding of calves was changed daily by new paddy straw.

### Recording of individual body weight

Birth weights of individual calves for the two groups were recorded soon after birth before feeding colostrums. Subsequently, individual body weights (kg) of calves under each group were recorded at weekly intervals from birth to the 9^th^ week of age. The calves were weighed in the morning before offering feed and water, with the help of digital weighing balance.

### Hematobiochemical parameters

The blood samples were collected within 6 h of birth and then at fortnightly interval prior to feeding and watering and were analyzed for hematological parameters, *viz*., Packed cell volume (PCV, %) and hemoglobin (Hb, g/dl). These parameters were estimated by using automatic hemanalyzer. Serum biochemical parameters, *viz*., Total serum protein (TSP, g/l) was determined by modified biuret method, using the kit (Span Cogent Diagnostics Ltd. India); albumin level (g/l) in serum was estimated by Bromocresol Green method, using the kit (Span Cogent Diagnostics Ltd. India). Globulin (g/l) was calculated by deducting albumin level from TSP and albumin globulin ratio (A:G) were estimated.

### Hormonal estimation

Important stress parameters namely T_3_ (ng/ml), T_4_ (ng/ml) and cortisol (ng/ml) were estimated by radio immunoassay using a gamma counter (Packard Bioscience Company, Model Cobra 11 Autogamma, USA).

### Meteorological observations

Environmental temperature (°C) and relative humidity (%) were recorded in the calf shed daily in morning between 7:30 a.m. and 8:00 a.m. and in evening between 3:30 p.m. and 4:00 p.m. For the external macroclimatic environment the data was collected from the Meteorological Station of Division of Physiology and Climatology, IVRI, Izatnagar.

### Statistical analysis

Data collected were analyzed by Statistical Analysis System (SAS, 2011) software programme, version 9.3. [14].

## Result

### Body weight changes and average daily gain (ADG)

The mean ± standard error (SE) of body weight (kg) changes and ADG (g) of calves of both groups are presented in Table-1. The effect of cold stress on body weight changes and ADG of calves in both groups was observed. The total body weight gain of 14.76±0.515 kg and ADG of 234.28±8.18 g was observed in calves of G_1_ as compared to total body weight gain of 20.38±0.514 kg and ADG of 323.49±8.16 g in case of G_2_. Thus, significant (p<0.01) increase in the total body weight gain and ADG was found in calves of G_2_ as compared to G_1_.

**Table-1:** Mean±SE of body weight (kg) changes and ADG (g) of Vrindavani calves.

Parameter	Group	Initial	Final	Total weight gain
Body weight (kg)	G_1_	20.6±2.01	35.36±2.43	14.76^A^±0.515
	G_2_	20.6±1.21	40.98±1.65	20.38^B^±0.514
ADG (g)	G_1_	-	234.28^A^±8.18	-
	G_2_	-	323.49^B^±8.16	-

ADG=Average daily gain, mean showing different superscript in upper case letters in a column differ significantly at 1% (p≤0.01)

### Hematological profile in calves

The Mean±SE of PCV percentage and Hb concentration of calves in both groups at fortnightly interval are presented in Table-2.

**Table-2:** Mean±SE of hematological parameters of Vrindavani calves at fortnightly interval.

Parameter	Group	Period (in days)				

		0 day	15 day	30 day	45 day	60 day
PCV (%)	G_1_	35.30±1.28	33.36±1.35	35.53±1.45	38.17^a^±0.86	41.00^A^±1.00
	G_2_	35.60±1.18	32.93±0.65	36.91±0.60	35.36^b^±0.41	35.88^B^±0.67
Hb (g/dl)	G_1_	10.91±0.14	10.33±0.24	11.46±0.14	12.62^A^±0.19	13.08±0.45
	G_2_	10.94±0.27	10.84±0.31	11.02±0.15	11.43^B^±0.18	12.26±0.15

PCV=Packed cell volume; Hb=Hemoglobin, mean showing different superscript in upper case letters in a column differ significantly at 1% (p≤0.01) and in lower case letters in a column differ significantly at 5% (p≤0.05), SE=Standard error

### PCV

The PCV values recorded were 35.30±1.28 and 35.60±1.18% for calves of G_1_ and G_2_, respectively at birth and no significant difference were found between these two values at the start of experiment. On 45^th^ day of the experiment, the PCV values recorded were 38.17±0.86 and 35.36±0.41% for calves of G_1_ and G_2_, respectively. This value of PCV for calves of G_1_ was significantly (p<0.05) higher than that of G_2_. Similarly, on 60^th^ day of the experiment, the PCV values recorded were 41.00±1.00 and 35.88±0.67% for calves of G_1_ and G_2_, respectively. This value of PCV for calves of G_1_ was significantly (p<0.01) higher than that of G_2_.

### Hb

The Hb concentrations recorded were 10.91±0.14 and 10.94±0.27 g/dl for calves in G_1_ and G_2_, respectively at birth. There was no significant difference between these two values at the start of the experiment. However, on 45^th^ day of the experiment, the Hb concentrations recorded were 12.62±0.19 and 11.43±0.18 g/dl for calves in G_1_ and G_2_, respectively. This value of Hb for calves of G_1_ was significantly (p<0.01) higher than that of G_2_.

### Serum biochemical profile in calves

The mean ± SE of Total serum protein (TSP, g/l), albumin (g/l), globulin, (g/l) and Albumin globulin ratio (A:G) of calves in both groups at fortnightly interval are presented in Table-3.

**Table-3:** Mean±SE of serum biochemical parameters of Vrindavani calves at fortnightly interval.

Parameter	Group	0 day	15 day	30 day	45 day	60 day
TSP (g/l)	G_1_	55.22±0.50	59.72±1.26	63.16±1.02	68.58^A^±1.28	70.00±0.69
	G_2_	55.54±0.78	58.70±0.82	62.10±0.34	63.58^B^±0.60	67.56±0.98
Albumin (g/l)	G_1_	26.48±0.31	30.78±0.99	33.46±1.42	36.20^A^±0.89	38.92^a^±0.88
	G_2_	27.34±0.58	28.94±0.44	32.42±2.15	31.98^B^±0.29	35.12^b^±0.73
Globulin (g/l)	G_1_	28.74±0.25	28.94±1.20	29.70±1.49	32.38±1.27	31.08±1.08
	G_2_	28.20±0.93	29.76±0.72	29.68±1.93	31.60±0.51	32.44±1.61
A:G	G_1_	0.92±0.01	1.07±0.07	1.15±0.11	1.12±0.06	1.26±0.07
	G_2_	0.97±0.05	0.98±0.03	1.14±0.18	1.01±0.02	1.10±0.08

TSP=Total serum protein, A:G=Albumin globulin ratio, mean showing different superscript in upper case letters in a column differ significantly at 1% (p≤0.01) and in lower case letters in a column differ significantly at 5% (p≤0.05), SE=Standard error

### TSP

The TSP levels in calves immediately after birth were 55.22±0.50 and 55.54±0.78 g/l for G_1_ and G_2_, respectively with no significant (p≥0.05) difference. However, comparatively higher values of TSP were found in calves of G_1_ as compared to G_2_ throughout the study. The TSP values for the G_1_ and G_2_ on 45^th^ day of the study were found to be 68.58±1.28 and 63.58 ±0.60 g/l, respectively showing a significant (p<0.01) difference between the groups.

### Albumin, globulin and A:G

The serum albumin concentrations recorded were 26.48±0.31and 27.34±0.58 g/l for the calves in G_1_ and G_2_, respectively at birth. For calves of G_1_ and G_2_, the values recorded were 36.20 ±0.89 and 31.98 ±0.29 g/l, respectively on 45^th^ day of the study showing a significant (p<0.01) difference between the groups. Similarly, on 60^th^ day of the experiment, the albumin values estimated were 38.92±0.88 and 35.12±0.73 g/l for calves of G_1_ and G_2_, respectively. This value of albumin for calves of G_1_ was significantly (p<0.05) higher than that of G_2_.

The values of serum globulin did not differ significantly between the groups. The serum globulin concentrations recorded were 28.74±0.25 and 28.20±0.93 g/l at birth and 31.08±1.08 and 32.44±1.61 g/l on 60^th^ day of study for the calves in G_1_ and G_2_, respectively. The values of A:G were found comparatively higher, although non-significant in calves of G_1_ as compared to G_2_.

### Hormonal parameters

The mean ± SE of cortisol (ng/ml), triiodothyronine (T_3_, ng/ml) and thyroxine (T_4_, ng/ml) of calves in both groups at fortnightly interval are presented in Table-4.

**Table-4:** Mean±SE of serum hormonal parameters in Vrindavani calves at fortnightly interval.

Parameter	Group	0 day	15 day	30 day	45 day	60 day
Cortisol (ng/ml)	G_1_	57.12±4.98	21.70^A^±1.44	7.40±1.28	5.96^A^±0.82	3.96±0.41
	G_2_	55.94±4.60	11.38^B^±0.93	6.90±1.41	3.94^B^±0.67	3.20±0.37
T_3_ (ng/ml)	G_1_	3.28±0.06	2.64^a^±0.07	2.68^a^±0.07	2.58^A^±0.12	1.27±0.26
	G_2_	3.28±0.16	1.85^b^±0.24	2.14^b^±0.16	1.84^B^±0.17	1.26±0.24
T_4_ (ng/ml)	G_1_	64.62±5.43	59.28^A^±3.99	52.62±3.03	60.74^A^±3.62	51.9±3.44
	G_2_	62.42±5.91	42.66^B^±1.74	48.30±3.09	42.78^B^±1.78	49.88±3.56

T_3_=Triiodothyronine, T_4_=Thyroxine, mean showing different superscript in upper case letters in a column differ significantly at 1% (p<0.01) and in lower case letters in a column differ significantly at 5% (p<0.05), SE=Standard error

### Cortisol

In general a trend in the level of cortisol was observed from birth to 60 days of age. At birth a very high value of cortisol was observed followed by decreased levels in both groups with increase in age. The mean serum concentrations of cortisol at birth were 57.12±4.98 and 55.94±4.60 ng/ml and 3.96±0.41and 3.20±0.37 ng/ml at 60^th^ day of the study in calves of G_1_ and G_2_, respectively. Comparatively higher values of cortisol were found in calves of G_1_ as compared to G_2_. The mean serum concentrations of cortisol differed significantly (p<0.01) on day 15 and day 45 of the experiment and were higher in calves of G_1_ than G_2_.

### Triiodothyronine (T_3_) and Thyroxine (T_4_)

The mean serum concentrations of T_3_ at birth were found higher in calves of both groups and were 3.28±0.06 and 3.28±0.16 ng/ml for calves of G_1_ and G_2_, respectively. In general, the values of T_3_ were found comparatively higher in calves of G_1_ as compared to G_2_ and the differences were significant (p<0.05) on 15 and 30^th^ day and highly significant (p<0.01) on 45^th^ day of the study. Similarly, the T_4_ concentrations were also high at birth in calves of both groups and values were 64.62±5.43 and 62.42±5.91 ng/ml in calves of G_1_ and G_2_, respectively. The T_4_ concentrations were significantly (p<0.01) higher on day 15 and day 30 in the calves of G_1_ as compared to G_2_.

## Discussion

### Body weight changes and ADG

The reduced rate of body weight gain in the calves of G_1_ might be due to cold-induced thermogenesis. This homeostatic response to cold stress might have increased heat production by using substrates mobilized from body tissues or from dietary metabolizable energy. Higher ADG in the calves of G_2_ as compared to G_1_ might be due to comfortable micro-environment provided by the infrared lamps. Due to the directional, draught free and instant heat properties of infrared radiations, the comfort zone for calves of G_2_ was achieved within a short period. On the other hand, conventional warm air systems usually take comparatively longer time to bring temperature to a comfort level. A more suitable environment for piglets was observed in the creeps equipped with infrared lamps compared to those with the thermal floor and incandescent light bulb [15]. Higher daily weight gain was also reported in calves provided with blankets than the calves with no such provision during winter [16]. Similarly, higher ADG although non-significant was observed in buffalo calves housed in conventional closed barn house as compared to calves housed in loose houses during winter [17].

### Hematological parameters

The reason for the increase in PCV and Hb in the calves of G_1_ might be due to increase in the synthesis of RBC and Hb to maintain the homeostasis. Higher values of PCV and Hb were also reported in cold stressed lambs compared to lambs protected against cold weather [18].

### Serum biochemical parameters

The calves in G_1_ showed comparatively higher concentrations of serum biochemical parameters than those in G_2_. Significantly (p<0.01) higher values of TSP and albumin found on day 45 in calves of G_1_ as compared to G_2_ might be due to their response to cold winter to maintain homeostasis. Higher values of plasma protein were also reported in dairy calves due to cold stress [19].

### Hormonal parameters

#### Cortisol

Various stress measurements have been described and it has been reported that changes in hormones such as cortisol and thyroxine are utilized in quantifying the response to stress [20]. At birth very high level of cortisol was observed in calves of both groups. This neonatal hypercortisolemia might be due to the hypersecretion of cortisol by fetal adrenals, which precedes and probably induces parturition. Significantly (p<0.01) higher values of cortisol were observed on day 15 and day 45 in calves of G_1_ as compared to G_2_. The increased level of cortisol in calves of G_1_ might be due to increase in lipolysis and utilization of brown adipose tissue for heat production. The higher values at day 15 could be due to cold stress or stress due to the absence of mother as also reported in Friesian calves [21]. Significantly (p<0.05) higher values of cortisol in Brahman’s calves were also observed after their exposure to cold [22]. Similarly, significantly (p<0.05) higher values of cortisol were also reported in cold stressed lambs as compared to protected lambs [18].

### Thyroid hormones

The level of thyroid hormones recorded did not exhibit a specific trend. The high levels of triiodothyronine and thyroxine at birth might be due to an early adaptation of the calf to the external environment. Significantly (p<0.01) higher values were also reported in zero to 7 days old buffalo calves [23]. The higher values of T_3_ and T_4_ in calves of G_1_ might be due to the effort of calves to adapt their metabolic balance to cold conditions. Significant (p<0.05) increase in thyroid levels of Brahman calves were reported after birth when they were exposed to cold treatment [22]. Similarly, increased thyroid levels were also reported in rams exposed to cold weather [24].

### Economic importance of using infrared lamps

The cost of electricity per kilowatt-hour at the time of the study was five rupees. The infrared lamps (250 W) were used for about 16 h per day. The cost of utilization of electricity per day was therefore Rs. 20 (0.25 × 16 × 5) and for the whole period of study (63 days) was Rs.1260. The cost of infrared lamp was Rs. 250 and the installation cost per two calves was Rs. 50. Since infrared lamps were used at the rate of one per two calves. Hence total cost for two calves was Rs.1560. Therefore, additional cost of raising one calf during the study period was Rs.780 and hence the cost of rearing each calf per day was Rs. 12.38 (780/63). The newborn calves are usually susceptible to the cold during their early part of life. The climate in the Northern region of our country usually remains cold for 3 months. Therefore, additional cost of rearing of calves under infrared lamps for 3 months will be Rs.1114.20 (Rs.779.94 for 63 days) per calf. The age at sexual maturity mainly depends on body weight of animals. The age and body weight at sexual maturity of crossbred cattle is usually around 21 months (630 days) and 280 kg, respectively. It means body weight gain of 412 g per day (260/630, assuming birth weight of 20 kg) is required to reach the given body weight at the age of sexual maturity. The ADG was found significantly (p<0.01) higher in calves of G_2_ (323.49±8.16) as compared to calves of G_1_ (234.28±8.18). The extra body weight gain of 89.21 g per day was obtained in calves of G_2_ over the body weight gain of calves of G_1_. Therefore, extra body weight gain in calves of G_2_ over calves of G_1_ for 3 months would be 8.029 kg (5.62 kg for 63 days). One day decrease in productive life resulted in a loss to the tune of Rs. 368 in crossbred cows [25]. Hence, the expected gain of lifetime productive days in animals of G_2_ would be around 19.48 days (8.029/0.412) or 13.64 days (5.62/0.412 considering 63 days) as compared to calves of G_1_. This in turn would lead to total benefit of Rs. 7168.64 (19.48 × 368) and net benefit of Rs. 6388.64 (7168.64 – 1114.20) per calf considering 3 months or total benefit of Rs. 5019.52 (13.64 × 368) and net benefit of Rs.4239.58 (5019.52 – 779.94) per calf considering 63 days.

## Conclusion

The results of this study indicated that calves raised under the protection of economically viable infrared lamps had better growth rate and were in more comfortable conditions as compared to the calves maintained without the provision of infrared lamps. Thus, infrared lamps in the calf shed could be effectively used as one of the measures to ameliorate the adverse effects of cold during winter.

## Authors’ Contributions

SAB and BB have conceived, planned and designed the study. SAB, SAS, CT and ASG have conducted the research, analyzed and kept a due record of the data. Manuscript was framed and drafted by SAB, PB & KPJ under the aegis of BB. All authors read and approved the final manuscript.
